# Modeling the effect of in‐plane magnetic field gradients on asymmetric spin‐echo images with echo‐planar imaging readout

**DOI:** 10.1002/mrm.30625

**Published:** 2025-07-17

**Authors:** Philipp Wallimann, Patrick L. Y. Tang, Fatemeh Arzanforoosh, Stephanie Tanadini‐Lang, Nicolaus Andratschke, Marion Smits, Esther A. H. Warnert

**Affiliations:** ^1^ Department of Radiation Oncology University Hospital Zurich and University of Zurich Zurich Switzerland; ^2^ Department of Radiology & Nuclear Medicine Erasmus MC, University Medical Center Rotterdam Rotterdam The Netherlands; ^3^ Department of Radiotherapy Erasmus MC Cancer Institute, University Medical Center Rotterdam Rotterdam The Netherlands; ^4^ Brain Tumor Center Erasmus MC Cancer Institute, University Medical Center Rotterdam Rotterdam The Netherlands; ^5^ Department of Radiation Oncology University Medical Center Groningen Groningen The Netherlands

**Keywords:** asymmetric spin echo, echo‐planar imaging, magnetic field gradients, oxygen metabolism, qBOLD

## Abstract

**Purpose:**

To describe the impact of macroscopic magnetic field gradients (MFGs) in the phase‐encoding direction on MR images acquired with an asymmetric spin echo (ASE) sequence with echo‐planar imaging (EPI) readout.

**Methods:**

In EPI, the center of k‐space is read out at a shifted time point in the presence of phase‐encoding direction MFGs. The ASE signal equation was extended to account for a locally varying temporal offset $\tau_{\mathrm{eff}}$ between the spin echo and echo time due to MFGs. The impact on estimated quantitative blood oxygen level dependent (qBOLD) parameters was assessed using simulations. A $B_{0}$ map and ASE images with four different phase‐encoding directions and two different parallel‐imaging factors were acquired from 2 healthy volunteers. A robust linear regression was performed between the signal dependence on the phase‐encoding direction and MFGs calculated based on the $B_{0}$ map to test the derived signal equation.

**Results:**

Simulated qBOLD parameters were substantially modified by the local $\tau_{\mathrm{eff}}$. The volunteer images showed a logarithmic signal intensity ratio between images acquired with reversed phase‐encoding directions that showed a linear dependence on both the calculated MFGs in the phase‐encoding direction and the nominal temporal offset τ. The effect was strongly reduced for the images with the higher parallel‐imaging factor.

**Conclusion:**

The effects of phase‐encoding‐direction MFGs on volunteer ASE images is consistent with the proposed signal model and relevant for qBOLD measurements. This highlights the necessity to correct or mitigate in‐plane MFGs in ASE EPI, such as using a high parallel‐imaging factor.

## 1 INTRODUCTION

Blood deoxygenation and oxygen metabolism can be measured in vivo using MRI with blood oxygen level dependent (BOLD) contrast.^1^ A quantitative model describing the behavior of the MR signal in the presence of a blood vessel network was introduced by Yablonskiy et al.^2^ By acquiring MR images of the brain at multiple time points around a spin echo, this model enables the generation of spatially resolved maps of the reversible relaxation rate (${R_{2}}^{\prime }$), deoxygenated blood volume (DBV), and oxygen extraction fraction (OEF).^3^ The acquisition of these quantitative parameters, which characterize cerebral oxygen metabolism, is known as quantitative BOLD (qBOLD). Comprehensive reviews on this method can be found in Li et al.^4^ and in Alzaidi et al.^5^

OEF could offer value in characterizing a variety of pathologies.^6^ It is of particular interest in oncological imaging, as it can be combined with cerebral blood flow measurements to estimate the cerebral metabolic rate of oxygen consumption.^7^ This capability may aid in identifying tumor hypoxia,^8^ a condition linked to treatment resistance.^9^ Compared with hypoxia quantification methods such as polarographic oxygen electrodes or positron emission tomography, qBOLD has the advantage of being noninvasive and without radiation.^8^ Previous studies have demonstrated the potential of qBOLD parameters to distinguish different tumor microenvironments.^10^

In recent years, the acquisition of qBOLD information with an asymmetric spin echo (ASE) approach^11^ has gained increasing attention.^12^, ^13^, ^14^, ^15^, ^16^, ^17^, ^18^, ^19^, ^20^, ^21^ Repeated spin‐echo images are acquired, where the echo time (TE) is kept constant during every acquisition, but the 180° refocusing pulse is applied at time point TE/2−τ/2 for different selections of τ. This means that signals are acquired with time offset of τ from the time point where the signal is refocused. The advantage of this approach is the lack of $R_{2}$‐weighting because of the constant TE across acquisitions and the opportunity to apply a fast echo‐planar imaging (EPI) readout, which minimizes motion sensitivity.^12^

**FIGURE 1:**
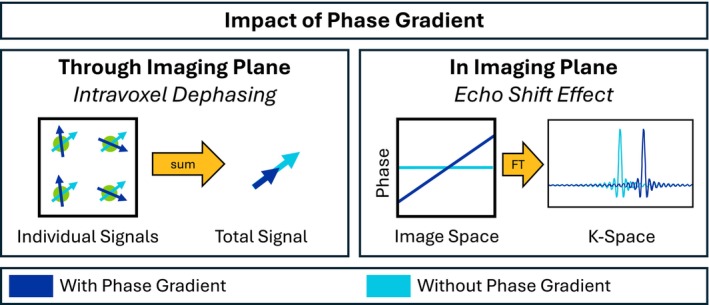
The impact of a phase gradient on the signal of a voxel. Left: Intravoxel dephasing leads to reduced magnitude of the summed signal. Right: Echo‐shift effect leads to a shift of the voxel's signal in k‐space, potentially resulting in a modified contrast. FT, Fourier transform.

A major concern for the accuracy of qBOLD data is the presence of susceptibility‐induced macroscopic magnetic field gradients (MFGs).^15^ The presence of an MFG leads to a gradient of the spin phases within a voxel, which is dependent on the time since the signal was refocused. For a two‐dimensional image, a phase gradient through the imaging plane causes intravoxel dephasing, thereby reducing the total signal of the voxel.^22^ A phase gradient in the two‐dimensional imaging plane results in a shift of the signal in k‐space. The center of k‐space is thus not read out at the nominal TE, but at a shifted time point, which potentially affects the image contrast. This effect is called echo shift effect.^23^ The two effects are illustrated in Figure 1.

Although previous ASE qBOLD publications have discussed and mitigated MFGs in slice direction,^14^, ^15^, ^16^, ^17^, ^18^ there has been limited emphasis on in‐plane MFGs. Blockley and Stone^15^ have discussed the relationship between the in‐plane resolution and signal dropout due to in‐plane MFGs. However, they did not quantitatively investigate echo‐shift effects below the dropout threshold. Similarly, Berman et al.^17^ have discarded voxels with high in‐plane MFGs but neglected the impact of weak in‐plane MFGs on the signal. Wood et al.^18^ have attempted to mitigate phase‐encoding direction MFGs using different pre‐phasing gradients in the phase‐encoding direction. Their signal curve as a function of τ showed unexpected maximums for τ≠0. The presence of uncorrected MFGs was hypothesized as a cause.

In this work, we provide a mathematical description of the effect of in‐plane MFGs on ASE images acquired with EPI readout. The echo‐shift effect results in a locally varying effective time offset $\tau_{\mathrm{eff}}$, which affects the ${R_{2}}^{\prime }$ and $R_{2}$ dependence of the observed signal. This provides a plausible explanation for a shifted signal peak as a function of τ, as observed in previous works.^18^, ^20^ Furthermore, we propose a method called simulated signal dropout to estimate $\tau_{\mathrm{eff}}$ from complex ASE images.

We demonstrate mathematically and through simulations that the echo‐shift effect in ASE images can result in biased estimation of qBOLD parameters. The *Python* code for the signal model and simulations is publicly available.

We validate the model using brain ASE images from healthy volunteers by demonstrating a linear relationship between the MFGs in the phase‐encoding direction of the EPI readout and the signal magnitude behavior when the phase‐encoding direction is reversed.

## 2 THEORY

### 2.1 Description of phase gradient in a voxel

The gradient of the phase inside a voxel at a given time point t from the spin echo under the effect of locally occurring MFGs $\vec{G}$ in three directions results in a k‐space shift of the signal of

(1) $$\vec{k}\left(t\right)=\gamma \;\vec{G}\;t+\vec{k_{0}},$$

where γ is the gyromagnetic ratio of protons, and $\vec{k_{0}}$ is the phase gradient occurring during the spin echo. This formulation adds the parameter $\vec{k_{0}}$ to the one presented by Reichenbach et al.^23^ $\vec{k_{0}}$ may be affected by a preparation gradient pulse (e.g., in a y‐shimming experiment^24^) or may appear unintentionally (e.g., due to eddy‐currents^25^). Note that x, y, and z describe the frequency‐encoding, phase‐encoding and slice directions, respectively.

### 2.2 Echo shift due to magnetic‐field gradients in EPI

The echo‐shift effect leads to a difference between the nominal TE and the actual readout timepoint of k‐space center.^23^ For EPI images, the duration of the readout in the frequency‐encoding direction is very short, of order 0.5ms; thus, an echo shift in the frequency‐encoding direction would have a minor effect on image contrast and we neglect it for the remainder of this work. The EPI echo train in the phase‐encoding direction can be interpreted as a long gradient‐echo readout,^24^ of order 20−100ms, which means an echo shift in the phase‐encoding direction can affect the contrast substantially.

For an ASE‐EPI experiment with a nominal offset of τ between the spin echo and TE, the echo‐shift effect in the presence of an MFG $G_{y}$ in the phase‐encoding direction leads to an effective time offset between the spin echo and the readout of the k‐space center of

(2) $$\tau_{\mathrm{eff}}\left(\tau \right)=\frac{\tau -\frac{k_{0,y}}{v}}{1+\frac{\gamma \;G_{y}}{v}}.$$

**FIGURE 2:**
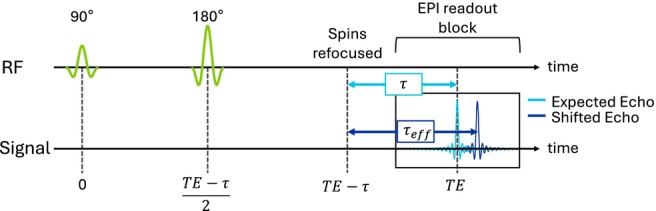
Sequence diagram for an asymmetric spin‐echo sequence with an echo‐planar imaging (EPI) readout. Echo time (TE) has a time offset of τ to the spin echo. The presence of a magnetic field gradient shifts the echo in k‐space and results in an effective time offset $\tau_{\mathrm{eff}}$ to the spin echo. RF, radiofrequency.

The derivation of Eq. (2) as well as the equations in the following subsections is provided in the Supporting Information. The value of v represents the k‐space velocity of the EPI train in the phase‐encoding direction. It can be noted that $\tau_{\mathrm{eff}}⟶\tau$ for v⟶∞, and $\tau_{\mathrm{eff}}⟶\tau -k_{0,y}/v$ for $G_{y}⟶0$. An illustration of $\tau_{\mathrm{eff}}$ on the sequence diagram is shown in Figure 2.

### 2.3 ASE signal model with echo shift

Using a similar notation as used by Cherukara et al.,^26^ the signal intensity S for a voxel in an ASE image as a function of the applied offset τ is given by

(3) $$S\left(\tau \right)=S_{0}\;\exp\left(-R_{2}\;\mathrm{TE}\right)\;f\left(\tau \right),$$

Where $S_{0}$ represents the baseline signal; f describes the signal behavior around the spin echo; and S(τ) is modeled as a single compartment.

If we consider the echo shift, the actual center of *k*‐space is read out at a time $\mathrm{TE}+\tau_{\mathrm{eff}}-\tau$ after the initial excitation and a time $\tau_{\mathrm{eff}}$ after the spin echo. Accounting for this in Eq. (3) leads to

(4) $$\begin{array}{ll} S_{\text{shift}}\left(\tau \right) & =S_{0}\;\exp\left(-R_{2}\;\mathrm{TE}\right)\;\exp\left(-R_{2}\;\left(\tau_{\mathrm{eff}}-\tau \right)\right)\;f\left(\tau_{\mathrm{eff}}\right)\;\\ & =S_{0}\;\exp\left(-R_{2}\;\mathrm{TE}\right)\;\exp\left(-R_{2}\;\frac{-\frac{\gamma \;G_{y}}{v}\tau -\frac{k_{0,y}}{v}}{1+\frac{\gamma \;G_{y}}{v}}\right)\;\\ & \;\times f\left(\frac{\tau -\frac{k_{0,y}}{v}}{1+\frac{\gamma \;G_{y}}{v}}\right), \end{array}$$

Where the signal dependence on τ is no longer given only by f, but it is also contaminated by an $R_{2}$ decay. That means that direct measurement of f is not possible in the presence of an MFG in phase‐encoding direction when using an EPI readout in an ASE experiment.

This expression does not consider through‐plane dephasing, but that can be done with an additional multiplicative factor.^13^, ^17^, ^22^

### 2.4 Impact of echo shift on qBOLD measurements

In qBOLD, f describes the signal behavior of a blood vessel network in the static dephasing regime,^2^ as given by $f\left(t\right)=\exp\left(-\mathrm{DBV}\int_{0}^{1}\frac{\left(2+u\right)\sqrt{1-u}}{3\;u^{2}}\left(1-J_{0}\left(\frac{3}{2}\;\delta \omega \;t\;u\right)\right)\mathrm{du}\right)$, where $J_{0}$ is the zero‐order Bessel function; DBV is the fractional deoxygenated blood volume; and $\delta \omega =\frac{4}{3}\pi \;\gamma B_{0}∆\chi_{0}\mathrm{Hct}\;\mathrm{OEF}$, where $B_{0}$ is the field strength of the primary magnetic field, $∆\chi_{0}$ is the susceptibility difference between the tissue and deoxyhemoglobin, and Hct is the fractional hematocrit.

This f can be separated into two regimes, where the function can more easily be expressed analytically.^2^ For large |τ|, f shows linear‐exponential $R_{2}^{\prime }$ decay, which can be modeled with $f\left(t\right)=\exp\left(\mathrm{DBV}-R_{2}^{\prime }\left|t\right|\right)$ with $R_{2}^{\prime }=\mathrm{DBV}\;\delta \omega$.^2^ We define $S_{\mathrm{lin}}$ as the signal function from Eq. (4) with this f. By including the echo‐shift effect, we can express the apparent $R_{2}^{\prime }$ in the linear exponential regime as follows:

(5) $$\bar{R_{2}^{\prime }}=\left\{\begin{array}{l} \;\frac{-R_{2}\frac{\gamma \;G_{y}}{v}+R_{2}^{\prime }}{1+\frac{\gamma \;G_{y}}{v}},\tau \geq \frac{k_{0,y}}{v}\\ \frac{R_{2}\frac{\gamma \;G_{y}}{v}+R_{2}^{\prime }}{1+\frac{\gamma \;G_{y}}{v}},\tau <\frac{k_{0,y}}{v} \end{array}\right.$$

Thus, $R_{2}^{\prime }$ derived based on the asymptotic behavior at high τ values may be affected by even small MFGs in phase‐encoding directions. For $\gamma \;G_{y}/v⟶0$, we observe $\bar{R_{2}^{\prime }}\to R_{2}^{\prime }$.

For small |τ|, the function f can be approximated with the quadratic‐exponential function $f\left(t\right)=\exp\left(-0.3\;\mathrm{DBV}\;{\left(\delta \omega \;t\right)}^{2}\right)$.^2^ We define $S_{\text{quad}}$ as the signal function from Eq. (4) with this f. In the log‐linear qBOLD model,^26^ the signal trajectory from the linear exponential regime is extrapolated to τ=0ms, and the DBV is estimated as $\mathrm{DBV}=\ln\left(S_{\mathrm{lin}}\left(0\right)/S_{\text{quad}}\left(0\right)\right)$.

By including the echo‐shift effect for $S_{\mathrm{lin}}$ and $S_{\text{quad}}$, we can derive an expression for an apparent DBV, which takes the following form:

(6) $$\bar{\mathrm{DBV}}=\left\{\begin{array}{l} \mathrm{DBV}\left(1+\delta \omega \frac{\frac{k_{0,y}}{v}}{1+\frac{\gamma \;G_{y}}{v}}+0.3{\left(\delta \omega \;\frac{\frac{k_{0,y}}{v}}{1+\frac{\gamma \;G_{y}}{v}}\right)}^{2}\right),0\geq \frac{k_{0,y}}{v}\\ \mathrm{DBV}\left(1-\delta \omega \frac{\frac{k_{0,y}}{v}}{1+\frac{\gamma \;G_{y}}{v}}+0.3{\left(\delta \omega \;\frac{\frac{k_{0,y}}{v}}{1+\frac{\gamma \;G_{y}}{v}}\right)}^{2}\right),0<\frac{k_{0,y}}{v} \end{array}\right.$$

It is worth noting that $\bar{\mathrm{DBV}}⟶\mathrm{DBV}$ for $k_{0,y}/v⟶0$.

Because δω is proportional to OEF, and the $R_{2}$ component in $\bar{R_{2}^{\prime }}$ is generally unknown, it may be difficult to estimate a correct OEF from the apparent values $\bar{\mathrm{DBV}}$ and $\bar{R_{2}^{\prime }}$.

### 2.5 Interaction between echo shift and phase‐encoding direction

The echo shift is sensitive to the MFG polarity,^23^ which is apparent by the dependence of $\tau_{\mathrm{eff}}$ on the sign of $G_{y}$. This allows us to design an experiment to investigate the impact of the echo‐shift effect on the signal magnitude. If two ASE‐EPI images are acquired with reverse phase‐encoding directions, we can interpret that as two images subject to opposite $G_{y}$. The term $\tau_{\mathrm{eff},\mathrm{rev}}$ is introduced to denote the $\tau_{\mathrm{eff}}$ observed for the image with reversed phase encoding.

For the linear exponential regime of f and τ>0, we would then expect the natural logarithm of the ratio of signal intensity between the opposing phase encoding directions, S and $S_{\mathrm{rev}}$, to follow the relationship

(7) $$\ln\left(\frac{S_{\mathrm{lin}}\left(\tau \right)}{S_{\mathrm{lin},\mathrm{rev}}\left(\tau \right)}\right)=-\left(R_{2}^{\prime }+R_{2}\right)\cdot \left(\tau_{\mathrm{eff}}-\tau_{\mathrm{eff},\mathrm{rev}}\right)$$

If the $\tau_{\mathrm{eff}}$ terms are split up into their components, the dependence on $G_{y}$ can be expressed as

(8) $$\ln\left(\frac{S_{\mathrm{lin}}\left(\tau \right)}{S_{\mathrm{lin},\mathrm{rev}}\left(\tau \right)}\right)=2\left(R_{2}^{\prime }+R_{2}\right)\;\tau \;\frac{\frac{\gamma \;G_{y}}{v}}{1-{\left(\frac{\gamma \;G_{y}}{v}\right)}^{2}}$$

The validity of Eqs. (7) and (8) can be tested in an experiment to demonstrate the existence of the echo shift effect in ASE images.

## 3 METHODS

### 3.1 Simulations

#### 3.1.1 Implementation

A simulation of the ASE‐EPI signal behavior as described in Eq. (4), and with a numerical calculation of f describing a blood vessel network in the static dephasing regime,^2^ has been implemented in *Python* (Python Software Foundation). The simulation also models through‐plane dephasing and in‐plane signal dropout. The code is publicly available at https://github.com/Waphil/ASE_MFG_simulation.

#### 3.1.2 Parameters

Unless otherwise specified, the tissue simulations reported in this work intend to represent healthy gray matter,^26^, ^27^ and the following tissue parameters are used: γ=267.51radMHz/T, $∆\chi_{0}=0.264\;\mathrm{ppm}$, Hct=0.4, OEF=0.4, DBV=0.03, $R_{2}=10\;\mathrm{Hz}$, $S_{0}=1.$ These parameters fall within the parameter ranges of previous simulation studies.^26^, ^27^

The simulated hardware and sequence parameters were chosen in accordance with the volunteer experiment described in Section 3.2. This meant the following parameter choices: TE=74ms, ∆y=3.5mm, ∆z=2.9mm, number of phase encoding steps $n_{p}=64$, v=0.077rad/(mmms). In all cases, a range of τ from −50 to +50ms was simulated as 10 000 discrete points.

#### 3.1.3 MFG impact on signal behavior

The signal as a function of τ was simulated for 11 equidistant values of $G_{y}$ from −50 to +50μT/m, which we refer to as the baseline scenario. Such values of $G_{y}$ can be expected in brain imaging at 3 T.^15^ We additionally simulated the same trajectories with $R_{2}=0\;\mathrm{Hz}$ (i.e., no $R_{2}$ decay), with a doubled v and with two different k‐space shifts $k_{0,y}=\pm 0.5\;\mathrm{rad}/\mathrm{mm}$.

#### 3.1.4 y‐Shimming impact on signal behavior

The signal as a function of τ was simulated for 11 equidistant values of $k_{0,y}$ from −0.90 to +0.90rad/mm, which we refer to as the baseline scenario. These values of $k_{0,y}$ represent the range that do not cause signal dropout^15^ at τ=0ms and $G_{y}=0\;\mathrm{\mu T}/m$. We additionally simulated the same trajectories with $R_{2}=0\;\mathrm{Hz}$ (i.e., no $R_{2}$ decay), with a doubled v and with two different MFGs $G_{y}=\pm 50\;\mathrm{\mu T}/m$.

In each case, the root mean squared value across all trajectories is calculated to simulate the image combination procedure used for different y‐shimmed images by Wood et al.^18^

#### 3.1.5 Echo‐shift impact on log‐linear qBOLD parameters

Based on the simulated signal trajectories, we simulate the log‐linear qBOLD approach^16^, ^26^ by fitting a linear exponential curve to the signals simulated in the range of τ from 45 to 50ms using a least‐squares fit. This range of τ was chosen to ensure that the signal was in the linear exponential regime in all simulated scenarios. The exponent of this curve gives an estimation for ${R_{2}}^{\prime }$. This curve was extrapolated to τ=0ms, and the difference between the extrapolated mono‐exponential signal at τ=0ms and the simulated signal at τ=0ms was used to estimate DBV. Based on ${R_{2}}^{\prime }$ and DBV, OEF was calculated.

The signal trajectories were created with identical settings as described in Section 3.1.2.

We performed the simulation for 1000 values of $\gamma G_{y}/v$ between −0.3 and 0.3 and repeated that procedure for 11 equidistant values of $k_{0,y}$ from −0.90 to +0.90rad/mm. The values of $\gamma G_{y}/v$ were simulated with a constant v=0.077rad/(mmms) and varying $G_{y}$ from −86 to +86μT/m. We show the resulting apparent qBOLD parameters ${R_{2}}^{\prime }$, DBV, and OEF. To contextualize the $\gamma G_{y}/v$ values, we visually highlight the region corresponding to $G_{y}$ between −11.9 and +10.0μT/m with the simulated settings. At 3 T, 50% of the voxels in the cortex have an anterior–posterior direction MFG in this range, according to Blockley et al.^15^

### 3.2 Volunteer experiments

#### 3.2.1 Data acquisition and postprocessing

To analyze the impact of MFGs on ASE images in vivo, 2 healthy volunteers (1 female, 1 male, ages 36 and 28 years) were recruited under local ethical approval (NL85764.078.23). Brain MR images were acquired of the volunteers using a 3T whole‐body MRI scanner (Signa Premier; GE, Chicago, IL, USA). For the ASE images of the volunteers, an inversion pulse is applied to suppress CSF signal. This approach is called fluid‐attenuated inversion‐recovery ASE (FLAIR‐ASE),^20^ and here “FASE” for the sake of brevity. Four different phase‐encoding directions were acquired with the same field of view. This means that the echo‐shift behavior was investigated along two pairs of opposing phase‐encoding direction (posterior–anterior/anterior–posterior and right–left/left–right), which allows a validation of the signal predictions described in Section 2.5. Additionally, a subset of τ values was acquired for the phase‐encoding directions right‐left/left‐right with doubled parallel‐imaging factor to investigate the dependence of the signal behavior on the k‐space velocity. FASE images were reconstructed as magnitude and phase images. Additionally, a $B_{0}$ map was acquired using a dual‐echo gradient‐echo approach. The acquisition parameters are given in Table 1.

**TABLE 1:** Acquisition parameters for the images in the volunteer experiment.

	FASE		$B_{0}$ map
TR[ms]	8000		100
TI[ms]	2000		–
TE[ms]	74		3.33, 5.81
$\mathrm{FOV}\;\left[{\mathrm{mm}}^{2}\right]$	224 × 224		240 × 240
Acquisition matrix	64 × 64		128 × 128
Slice thickness [mm]	2.9		2.9
Number of slices	36		50
Flip angle 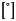	90		10
Receiver bandwidth (readout) [Hz/px]	1938		488
Averages	4 per τ and phase‐encoding direction (1 dummy, 3 averaged)		1
Parallel‐imaging factor (in PED)	2	4	2
$t_{\text{effES}}\;\left[\mathrm{ms}\right]$	0.354	0.179	–
PED	PA, AP, RL, LR	RL, LR	LR
τ[ms]	0,6,12,18,24,30,36,42,48	0,12,24,36,48	–

*Note*: For parallel‐imaging factor of 4, only a subset of the phase‐encoding directions and τ values were obtained.

Abbreviations: AP, anterior–posterior; FASE, fluid‐attenuated inversion‐recovery asymmetric spin echo; FOV, field of view; LR, left–right; PA, posterior–anterior; PED, phase‐encoding direction; RL, right–left; TE, echo time; TI, inversion time; TR, repetition time.

**FIGURE 3:**
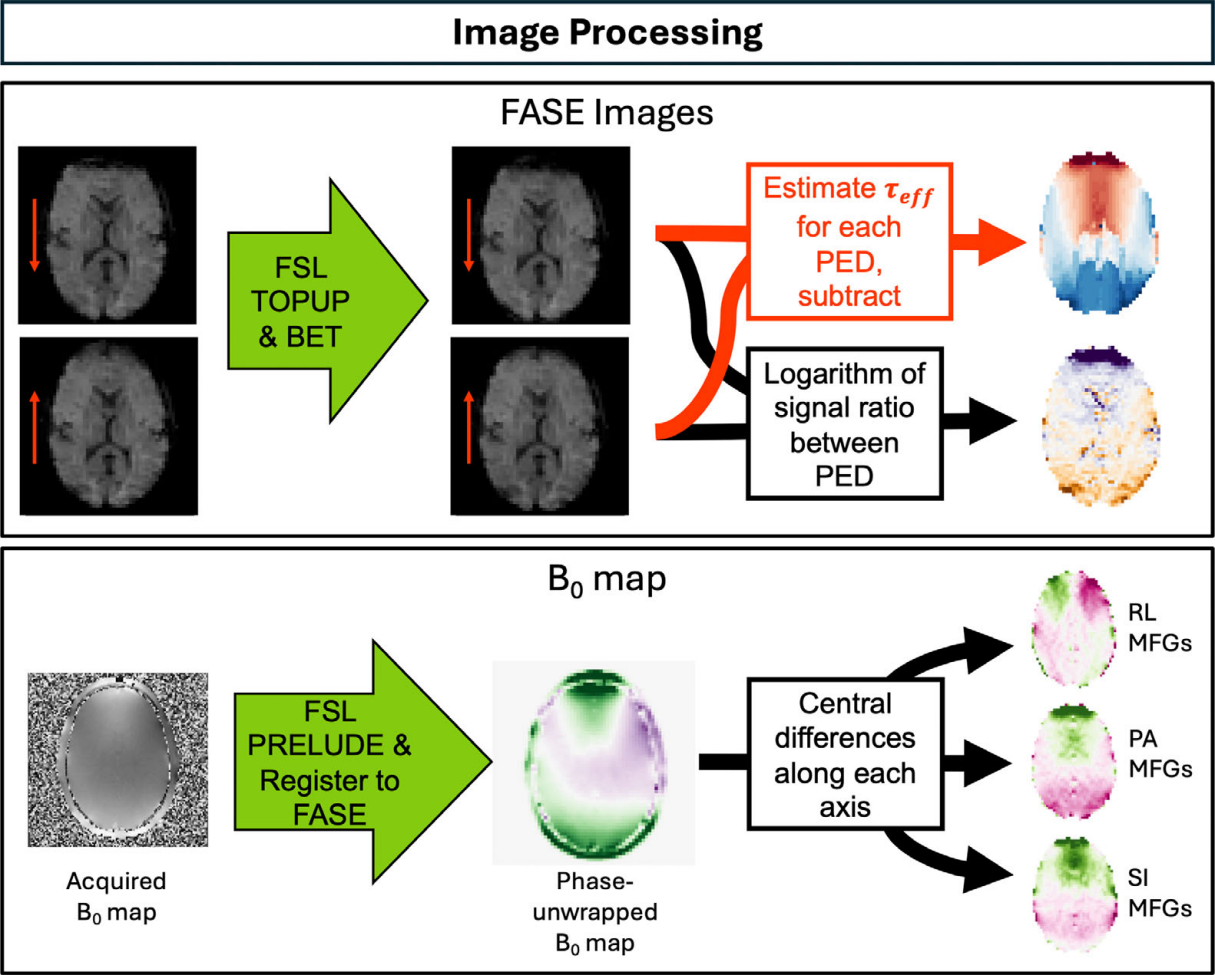
Image‐processing steps. For fluid‐attenuated inversion‐recovery asymmetric spin echo (FASE) images: Images are shown for a single τ, but processing is performed for each τ. The red arrows indicate the phase‐encoding directions. TOPUP distortion correction was performed, and the brain was extracted using FSL BET. The value of $\tau_{\mathrm{eff}}$ was estimated at every voxel from complex image data using the simulated signal dropout method. For $B_{0}$ map: Reconstructed off‐resonance map was unwrapped using FSL *PRELUDE*, then registered and resampled to the FASE image coordinates. The magnetic field gradients (MFGs) were calculated along each axis using central differences. PED, phase‐encoding direction; PA, posterior–anterior; RL, right–left; SI, superior–inferior.

The image postprocessing is visualized in Figure 3. For the FASE data, the relative motion of the individual acquired images was corrected by rigidly registering all the τ≠0ms images to the τ=0ms image using FLIRT^28^, ^29^ from FSL.^30^ The image distortion was corrected using the function TOPUP^31^ from FSL. For each pair of opposing phase‐encoding directions (anterior–posterior/posterior–anterior and left–right/right–left), the distortion was estimated based on the τ=0ms images, because they are the least affected by signal dropout and subsequently applied to images of all τ values with those two phase‐encoding directions. The Jacobian modulation option for TOPUP was chosen, because that method does not mix the contrast of the images between the two different phase‐encoding directions.

From the distortion‐corrected τ=0ms images, the brain region was determined using BET^32^ from FSL and used as a region of interest for all further analyses.

Signal dephasing due to through‐plane MFGs was not corrected in our experiment.

#### 3.2.2 MFG calculation

The acquired $B_{0}$ map was phase‐unwrapped using PRELUDE from FSL. The image was then rigidly registered to the FASE images using FLIRT from FSL and trilinear interpolation. The MFGs in the different directions (i.e., right–left, posterior–anterior, and superior–inferior) were then calculated using central differences.^17^ For a given voxel in the FASE coordinates, this yields an estimated $G_{y}$ for a known phase‐encoding direction.

#### 3.2.3 Echo‐shift estimation

**FIGURE 4:**
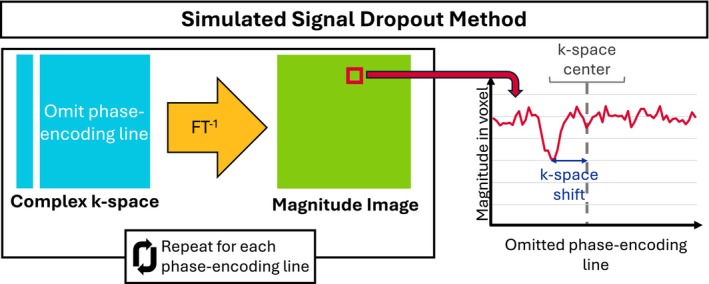
Simulated signal dropout method to determine k‐space shift per voxel. The method aims to identify shifts to the effective k‐space center caused by in‐plane macroscopic magnetic field gradients (MFGs). The phase‐encoding line of the complex k‐space data that leads to the minimum signal in a given voxel when omitted is interpreted to be the center of shifted k‐space in that voxel. The shift can be converted into $\tau_{\mathrm{eff}}$, given the known readout timings. The red signal magnitude curve is smoothed with a Gaussian filter to increase the robustness of the minimum magnitude determination. FT, Fourier transform.

After the image processing, we estimated a spatially resolved map of $\tau_{\mathrm{eff}}$ based on complex FASE images (Figure 4). We obtained the complex k‐space data by applying the Fourier transform to the complex images. The k‐space location at which the signal maximum was read out was determined through sequentially setting each k‐space line in the phase‐encoding direction to zero and reconstructing the corresponding magnitude images. For a given voxel, the curve describing the signal magnitude as a function of the removed k‐space line index was investigated. A Gaussian filter with standard deviation of 2 was applied to reduce random noise in the curve. The index $i_{\min}$, where the signal was minimal, was identified and interpreted as the k‐space line that contains the shifted center of k‐space for that voxel, because the omission of the k‐space line caused the largest drop in signal magnitude.

Using a known center index of k‐space $i_{\text{center}}$, which corresponds to a time τ after the spin echo, and a known effective echo spacing $t_{\text{effES}}$ of the EPI readout, this k‐space index showing minimal signal $i_{\min}$ was converted into $\tau_{\mathrm{eff}}$ with the formula $\tau_{\mathrm{eff},\text{estimated}}=t_{\text{effES}}\left(i_{\min}-i_{\text{center}}\right)+\tau$. This procedure of estimating $\tau_{\mathrm{eff}}$ is referred to as the “simulated signal dropout” method.

For pairs of images acquired with opposite phase‐encoding directions but otherwise identical parameters, the two estimated $\tau_{\mathrm{eff}}$ were subtracted voxelwise. This yields an estimation of $\tau_{\mathrm{eff}}-\tau_{\mathrm{eff},\mathrm{rev}}$ for every voxel.

To align with the directions of the calculated MFGs, the directions left–right and anterior–posterior were considered the reversed phase‐encoding directions.

#### 3.2.4 Signal dependence on phase‐encoding direction

Likewise, for pairs of images with the same τ and opposite phase‐encoding direction, the ratio of the signal magnitudes was calculated, and the natural logarithm of it was computed. Thus, for every voxel we obtain a value for $\ln\left(S/S_{\mathrm{rev}}\right)$.

#### 3.2.5 Data evaluation

Based on the theoretical calculations, we would expect a linear relationship between $\ln\left(S/S_{\mathrm{rev}}\right)$ and $\tau_{\mathrm{eff}}-\tau_{\mathrm{eff},\mathrm{rev}}$ for high τ values, as expressed in Eq. (7). Similarly, we would expect a near‐linear relationship between $\ln\left(S/S_{\mathrm{rev}}\right)$ and $G_{y}$, as expressed in Eq. (8).

We investigated the existence of these linear relationships by performing a robust linear regression with the Theil‐Sen approach^33^, ^34^ with intercept (*SciPy*,^35^ *Python*) between $\ln\left(S/S_{\mathrm{rev}}\right)$ and $\tau_{\mathrm{eff}}-\tau_{\mathrm{eff},\mathrm{rev}}$ for all voxel measurements obtained in the brain. The regression was performed separately for each volunteer, for each pair of phase‐encoding directions, for each parallel‐imaging factor and for each nominal τ. This enabled an analysis of the dependence of the linear slope on τ and k‐space velocity. The linear relationship between $\ln\left(S/S_{\mathrm{rev}}\right)$ and $G_{y}$ was analyzed in the same manner.

## 4 RESULTS

### 4.1 Simulations

**FIGURE 5:**
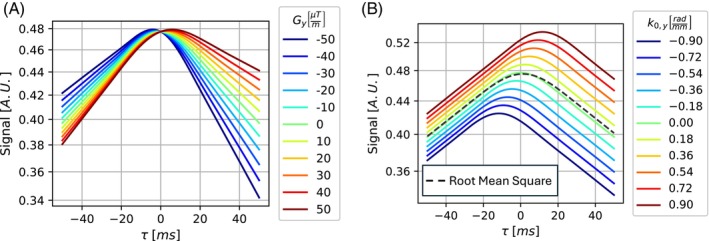
Simulated asymmetric spin‐echo signal for different offsets τ. Simulation parameters are provided in the text. The signal without echo shift is shown for $G_{y}=0$ & $k_{0,y}=0$. (A) Signal trajectories for different choices of phase‐encoding‐direction macroscopic magnetic field gradients $G_{y}$. (B) Signal trajectories for different choices of phase‐encoding‐direction baseline k‐space shifts $k_{0,y}$. The black dashed line represents the root mean square of all signals.

Simulated ASE signal trajectories as a function of offset τ are shown for the baseline scenarios in Figure 5. Additional simulation scenarios are shown in Figure S1.

#### 4.1.1 MFG impact on signal behavior

Phase‐encoding direction MFGs cause an asymmetry in the signal dependence on τ depending on the magnitude and sign of $G_{y}$ (Figure 5A). All curves coincide at τ=0, but they show different slopes for high |τ| depending on $G_{y}$. The asymmetry around τ=0 disappears if no $R_{2}$ decay occurs (Figure S1A); the impact of the $G_{y}$ is lower at higher k‐space velocity (Figure S1C); and the τ value where the curves coincide is dependent on $k_{0,y}$ (Figure S1E,F).

#### 4.1.2 y‐Shimming impact on signal behavior

Baseline k‐space shifts $k_{0,y}$ cause offset trajectories in the signal‐dependence on τ depending on the magnitude and sign of $k_{0,y}$ (Figure 5B). The slopes at high |τ| remain consistent across the scenarios. The root mean sum of squares of all trajectories shows slightly higher signal for τ>0 than the curve with $k_{0,y}=0$. If no $R_{2}$ decay occurs, the different trajectories show only an offset along τ (Figure S1B); the impact of the $k_{0,y}$ is lessened at higher k‐space velocity (Figure S1D); and the presence of a large $G_{y}$ results in signal trajectories demonstrating signal dropout in different regimes of τ (Figure S1G,H).

#### 4.1.3 Echo‐shift impact on log‐linear qBOLD parameters

**FIGURE 6:**
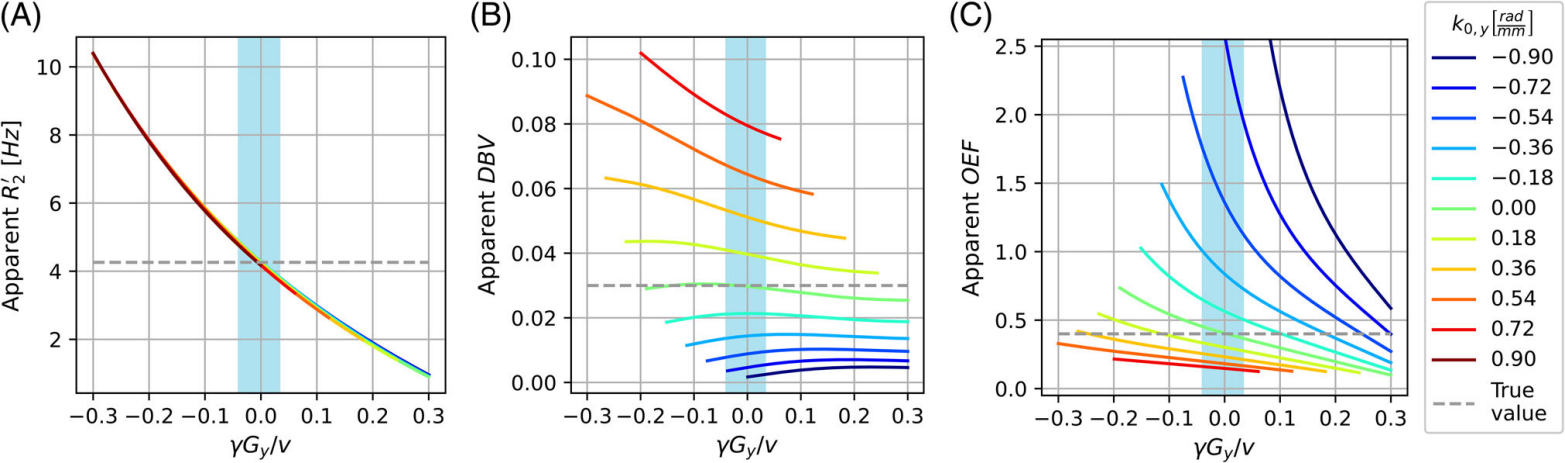
Apparent quantitative blood oxygen level–dependent (qBOLD) parameters based on log‐linear qBOLD model with simulated signal trajectories: ${R_{2}}^{\prime }$ (A), deoxygenated blood volume (DBV) (B), oxygen extraction fraction (OEF) (C). The legend of (C) applies to all subplots. Gaps in lines are a result of signal dropout and subsequent failure to apply the log‐linear qBOLD approach. The light blue shaded area represents the interval of $G_{y}$ between −11.8 and +10.0μT/m with the simulated settings, which has been reported to include 50% of the voxels in the cortex for the anterior–posterior direction.

The apparent qBOLD parameters resulting from applying the log‐linear qBOLD approach to simulated signal trajectories show large deviations from the true values in certain simulation scenarios (Figure 6). The ${R_{2}}^{\prime }$ values show a strong dependence on $\gamma G_{y}/v$ but a weak dependence on $k_{0,y}$ (Figure 6A). The DBV values show a strong dependence on $k_{0,y}$ and a moderate dependence on $\gamma G_{y}/v$ (Figure 6B). The OEF values are sensitive on both $\gamma G_{y}/v$ and $k_{0,y}$ (Figure 6C).

### 4.2 Volunteer experiments

**FIGURE 7:**
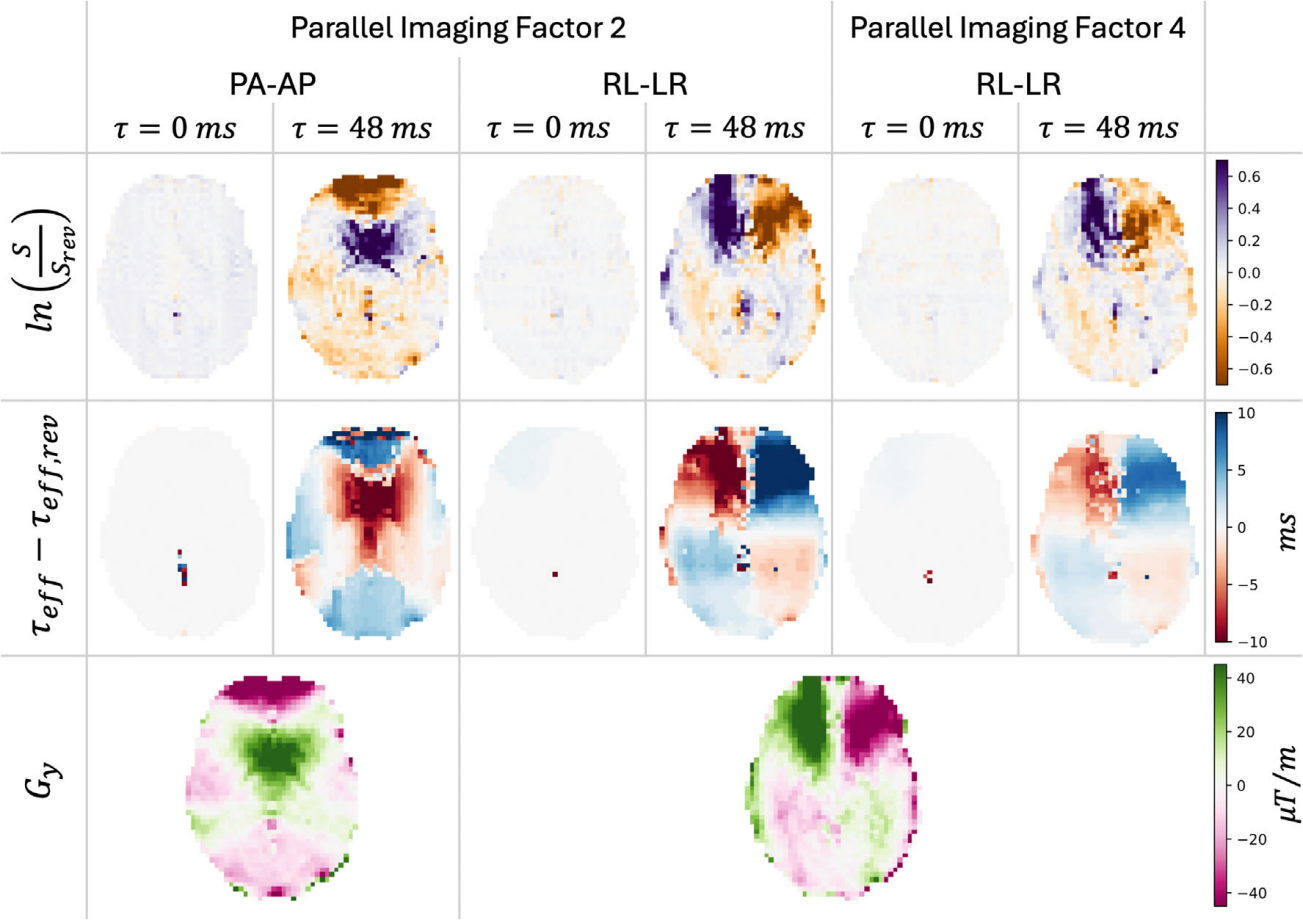
The quantities computed from the fluid‐attenuated inversion‐recovery asymmetric spin‐echo images and $B_{0}$ map shown on one slice for one of the volunteers. Both pairs of phase‐encoding directions, pairs of opposing phase‐encoding direction (PA‐AP and RL‐LR), are depicted for parallel‐imaging factor of 2, and the results from the phase‐encoding direction pair RL‐LR are additionally shown for parallel‐imaging factor of 4. The FASE quantities are shown on the τ=0ms and τ=48ms images. The color bars apply to all images in the row. AP, anterior–posterior; LR, left–right; PA, posterior–anterior; RL, right–left.

The three calculated quantities $\ln\left(S/S_{\mathrm{rev}}\right)$, $\tau_{\mathrm{eff}}-\tau_{\mathrm{eff},\mathrm{rev}}$ and $G_{y}$ are shown in one slice of the brain for Volunteer 2 in Figure 7. For the τ=0ms images, the $\ln\left(S/S_{\mathrm{rev}}\right)$ and $\tau_{\mathrm{eff}}-\tau_{\mathrm{eff},\mathrm{rev}}$ results show very small values that appear homogeneous in the brain, whereas for the τ=48ms image, they form heterogeneous patterns across the brain. Those patterns are similar in appearance to the heterogeneous patterns appearing in the calculated $G_{y}$. The parameters of the acquisitions with a parallel‐imaging factor of 4 showed substantially lower values of $\ln\left(S/S_{\mathrm{rev}}\right)$ and $\tau_{\mathrm{eff}}-\tau_{\mathrm{eff},\mathrm{rev}}$. A comparison of $\ln\left(S/S_{\mathrm{rev}}\right)$ in a slice across different τ is shown in Figure S2.

**FIGURE 8:**
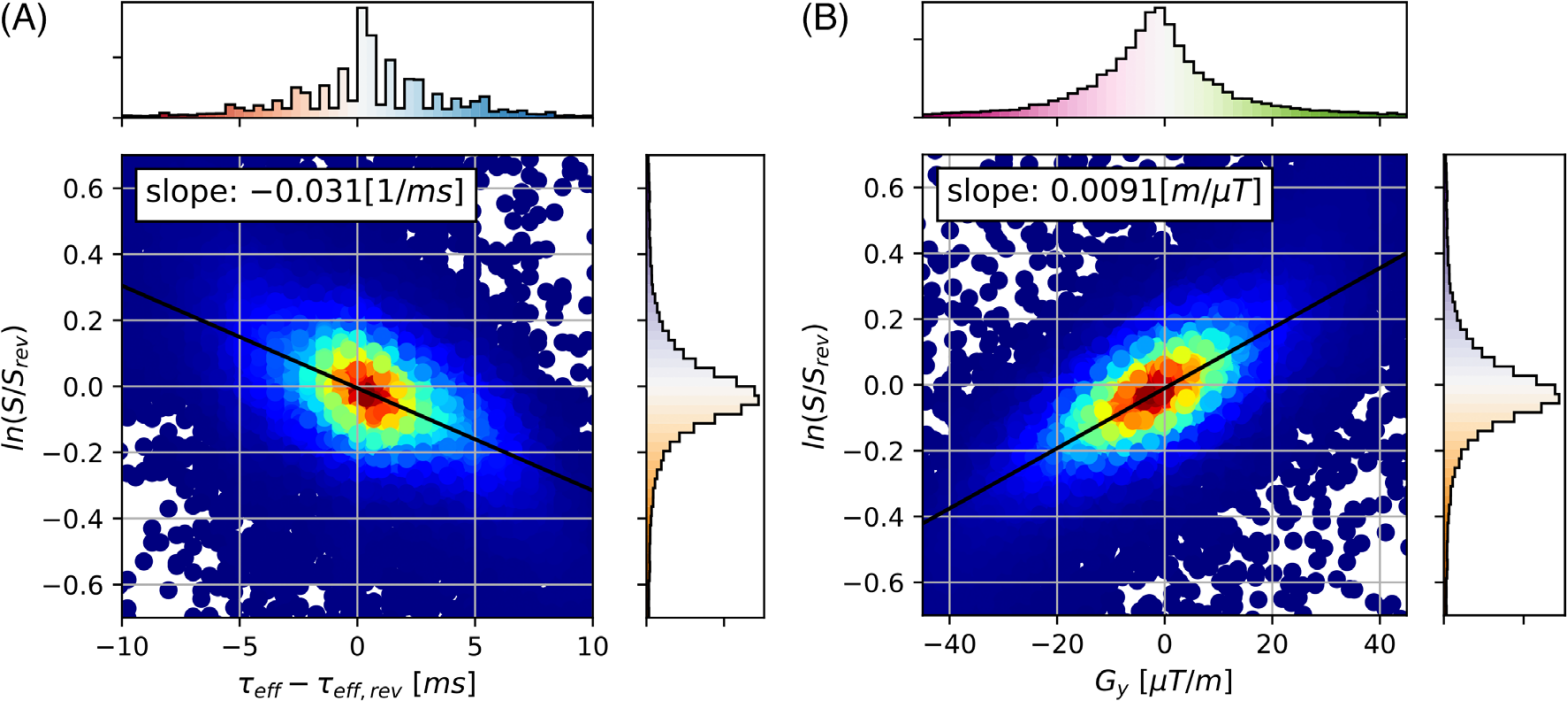
Scatter plots of the voxel‐wise relationship between the measured parameters in the brain. Data shown for Volunteer 2, τ=48ms, and phase‐encoding direction pair posterior–anterior/anterior–posterior. The linear fit between the variables is shown as a black line, and the numerical value of the slope is displayed. The histogram of the values on the x‐axis is shown above the scatter plot and of the values on the y‐axis on the right. The histogram colors correspond to the color bars shown in Figure 7. The scatter plot colors correspond to the density of points as determined using a two‐dimensional kernel density estimation. (A) relationship between $\ln\left(S/S_{\mathrm{rev}}\right)$ and $\tau_{\mathrm{eff}}-\tau_{\mathrm{eff},\mathrm{rev}}$. (B) Relationship between $\ln\left(S/S_{\mathrm{rev}}\right)$ and $G_{y}$.

**FIGURE 9:**
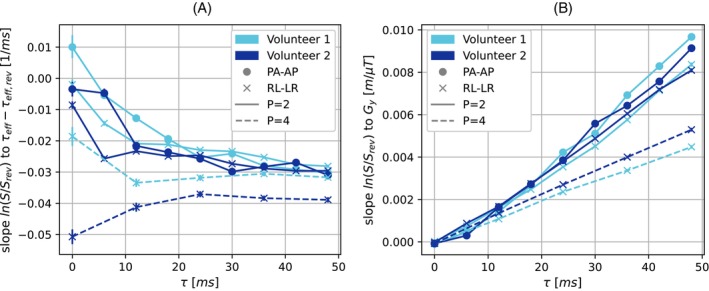
Dependence of linear slope between variables across all brain voxels on the nominally selected τ value. The colors of the lines correspond to the different volunteers, the markers to the different phase‐encoding direction pairs, and the line styles to the different parallel‐imaging factors. The whiskers show the 95% confidence interval, as provided by the robust linear regression. (A) Slope between $\ln\left(S/S_{\mathrm{rev}}\right)$ and $\tau_{\mathrm{eff}}-\tau_{\mathrm{eff},\mathrm{rev}}$. (B) Slope between $\ln\left(S/S_{\mathrm{rev}}\right)$ and $G_{y}$. AP, anterior–posterior; LR, left–right; PA, posterior–anterior; RL, right–left.

For Volunteer 2 for τ=48ms in the phase‐encoding directions posterior–anterior/anterior–posterior and parallel‐imaging factor of 2, the relationship between $\ln\left(S/S_{\mathrm{rev}}\right)$ and $\tau_{\mathrm{eff}}-\tau_{\mathrm{eff},\mathrm{rev}}$ is shown in Figure 8A, and between $\ln\left(S/S_{\mathrm{rev}}\right)$ and $G_{y}$ in Figure 8B. In each case, the linear function obtained with the robust linear regression is shown. For the slopes of the linear regressions between $\ln\left(S/S_{\mathrm{rev}}\right)$ and $\tau_{\mathrm{eff}}-\tau_{\mathrm{eff},\mathrm{rev}}$ (Figure 9A), we observe a decrease for τ values between 0ms and 24ms, and little variation for higher τ. The slopes are systematically lower for the parallel‐imaging factor of 4 images from Volunteer 2 than in all other configurations.

The slopes of the linear regressions between $\ln\left(S/S_{\mathrm{rev}}\right)$ and $G_{y}$ (Figure 9B) rise monotonously with τ at a consistent rate. The rise in slopes is systematically lower for the images acquired with parallel‐imaging factor of 4 compared to those acquired with parallel‐imaging factor of 2.

## 5 DISCUSSION

Our extension of previously established models for the echo‐shift effect^23^, ^24^ to ASE‐EPI images highlights challenges with their quantitative evaluation that have not been considered by existing research work. In‐plane MFGs can modify the signal dependence on the nominal time offset τ in ASE experiments, leading to a locally varying effective time‐offset $\tau_{\mathrm{eff}}$. This results in a dependence of the signal observed on $R_{2}$ and the local MFGs in the phase‐encoding direction. It is a potential explanation for previously reported anomalous results, such as a signal peak at τ≠0,^18^, ^20^ which we also observed in our simulations (Figure 5).

As demonstrated by our simulation results in Figure 6, the signal alterations due to MFGs in the phase‐encoding direction $G_{y}$ and baseline phase gradients in the phase‐encoding direction $k_{0,y}$ can have a substantial effect on the apparent qBOLD parameters derived from the images using a log‐linear qBOLD approach. Even small $G_{y}$ can have an effect on the ${R_{2}}^{\prime }$ estimation (Eq. (5) and Figure 6A), whereas DBV estimations are sensitive to $k_{0,y}$ (Eq. (6) and Figure 6B). Accurate qBOLD measurements using ASE with an EPI readout therefore require the mitigation or modeling of these effects.

Our volunteer experiment was able to confirm a different contrast between ASE images acquired with reversed phase‐encoding directions, which was expected with the echo shift effect. The phase‐encoding dependence of the signal magnitude at τ=48ms showed a similar pattern as the phase‐encoding direction MFGs and the estimated $\tau_{\mathrm{eff}}$ with the proposed simulated signal dropout method (Figure 7). The fact that the signal magnitude and estimated $\tau_{\mathrm{eff}}$ did not show this spatial heterogeneity for the τ=0ms image suggests that this heterogeneity does not arise from the image processing (e.g., faulty distortion correction).

The expected relationship between $\ln\left(S/S_{\mathrm{rev}}\right)$ and $\tau_{\mathrm{eff}}-\tau_{\mathrm{eff},\mathrm{rev}}$ in the linear exponential regime, outlined in Eq. (7), could be observed in practice for τ>20ms with a linear slope between the variables that is insensitive to τ and consistent across experiments (Figure 9A). The different behavior for τ<20ms may be related to the quadratic exponential regime and the fact that $\tau_{\mathrm{eff}}-\tau_{\mathrm{eff},\mathrm{rev}}$ would be smaller and thus uncertainties would affect the slope more. The systematically lower values for the parallel‐imaging factor 4 images from Volunteer 2 may be related to the interaction between the echo shift and through‐plane dephasing, which varies across images and was not corrected.

Similarly, the expected relationship between $\ln\left(S/S_{\mathrm{rev}}\right)$ and $G_{y}$, outlined in Eq. (8), was observed in the volunteer data with a linear slope between the variables that appears to be proportional to τ and inversely proportional to v (Figure 9B). This serves as evidence that our theoretical description of the echo‐shift effect applies in ASE images of the brain, and it demonstrates that the echo‐shift effect can be mitigated by acquiring ASE images with a higher parallel‐imaging factor.

### 5.1 Compensation strategies for echo‐shift effect

ASE images are assumed to have constant $R_{2}$ weighting across τ values due to the constant TE.^12^, ^13^ The echo‐shift effect introduces a dependence on $R_{2}$, as shown in Eq. (4), and thus should be mitigated or compensated.

The susceptibility of the signal to echo shift is directly affected by the k‐space velocity in the phase‐encoding direction. As a result, it is recommended to acquire ASE‐EPI images with a high k‐space velocity to mitigate the echo‐shift effect, such as by applying a high parallel‐imaging factor, which was demonstrated in this work, or using segmented EPI.

The application of y‐shimming^18^, ^24^ to mitigate phase‐encoding direction MFGs is not straightforward, as large k‐space shifts $k_{0,y}$ has a strong effect on $\tau_{\mathrm{eff}}$ and can therefore lead to substantially altered signal curves (Figure 5B). This alteration in turn complicates the fitting of DBV (Eq. (6) and Figure 6B), which may be a reason why Wood et al.^18^ reported that it was not possible to obtain an accurate fit of DBV simultaneously with ${R_{2}}^{\prime }$ in their experiment using y‐shimming. As a result, it is desirable to have $k_{0,y}$ near zero, which is expected if y‐shimming is avoided.

Because the observations about the signal behavior (Figure 9) are compatible with the described model, a numerical correction of the signal intensities based on the model may be possible. If $\tau_{\mathrm{eff}}$ has been estimated for each voxel, such as using the simulated signal dropout method or with Eq. (2) using a $G_{y}$ measurement, then a correction factor $C\left(\tau \right)=\exp\left(R_{2}\;\left(\tau_{\mathrm{eff}}-\tau \right)\right)$ can be multiplied to the signal. The only dependence of the corrected signal on $\tau_{\mathrm{eff}}$ would then be through $f\left(\tau_{\mathrm{eff}}\right)$. To determine C, the values for $R_{2}$ would have to be at least estimated. More details are provided in the Supporting Information.

If images with opposing phase‐encoding directions were acquired, calculating the geometric mean of the signals in opposing phase‐encoding direction partially cancels out the echo‐shift effect, as we demonstrate in Eq. (S3) of the Supporting Information. The dependence of the signal terms on $\gamma G_{y}/v$ would then be of second order or higher, which may be a sufficient mitigation for practical purposes.

Future publications of ASE‐EPI results should report enough details on the EPI readout such that the phase‐encoding direction and k‐space velocity in the phase‐encoding direction can be obtained. That way, the magnitude of the echo‐shift effect can at least be estimated.

An appropriate compensation of in‐plane MFGs could facilitate the acquisition of qBOLD data for clinical purposes with less quantitative bias, which for instance may help with previously reported inconsistent results of correspondence between OEF and hypoxia in brain tumors.^8^

### 5.2 Limitations

The presented echo‐shift model is particularly relevant for ASE EPI due to the low k‐space velocity in the phase‐encoding direction; thus, we only explored it in that context. However, the echo‐shift effect may also show up in a gradient‐echo sampling of spin‐echo experiment, where the effective TE of readouts far from the spin echo could be shifted due to in‐plane MFGs. For acquisition strategies in which multiple EPI readouts are acquired per excitation,^21^, ^36^ the individual impact of the echo shift on each readout could be modeled.

Previous works have discussed systematic differences between qBOLD parameter estimations originating from different acquisition strategies, with ASE overestimating DBV.^27^ The echo‐shift model does not offer a clear explanation for that, as the apparent DBV is not strongly dependent on $G_{y}$, as shown in Figure 6B. Apparent DBV is sensitive to $k_{0,y}$, but this should result in both increases and decreases of DBV if both positive and negative $k_{0,y}$ can appear. The role of the echo shift in systematic measurement errors in ASE qBOLD could be explored in future investigations.

In this work, we demonstrate the ability to mitigate the echo‐shift effect by using a higher parallel‐imaging factor. In the future, additional strategies for mitigation or correction could be systematically compared to establish an optimized processing workflow for ASE EPI.

## 6 CONCLUSION

We propose the echo‐shift model to account for MFGs in the phase‐encoding direction when acquiring ASE images with EPI readout. Our simulations demonstrate that the echo shift could explain previously reported anomalies in the ASE signal dependence on the temporal offset τ between the spin echo and TE. The simulations furthermore suggest that the echo shift effect has a large impact on estimated qBOLD parameters and thus needs to be mitigated. Our experiment with brain ASE images of volunteers demonstrates that the dependence of the image contrast on the phase‐encoding direction is consistent with the model of a shifted echo due to phase‐encoding MFGs. The effect could be mitigated by increasing the parallel‐imaging factor. The incorporation of the echo‐shift model into future qBOLD studies with ASE EPI may improve the accuracy of the parameter estimates.

## Supporting information


**Data S1.** Supporting information.

## Data Availability

A *Python* implementation of the echo‐shift simulation for ASE‐EPI images, along with an implementation of the simulated signal dropout method, are available at https://github.com/Waphil/ASE_MFG_simulation. For this work, the version with SHA‐1 hash d2a2a3c73634067b 3f269f4178934cd14ac44bdb was used.
