# Supplementary material for: Modeling the effect of in‐plane magnetic field gradients on asymmetric spin‐echo images with echo‐planar imaging readout
Source: Magn Reson Med. 2025 Jul 17;94(5):2086–99. doi: 10.1002/mrm.30625 (PMC12393211; doi:10.1002/mrm.30625)
Supplement: Supplementary file 1 — Data S1. Supporting information. [file MRM-94-2086-s001.pdf]

Supporting Information

Formulas and Derivations

Derivation of Eq. (2)

This is the derivation of the time point the center of k-space is read out in an ASE experiment under the influence of an MFG in phase encoding direction.

Let us neglect MFGs in frequency-encoding and slice direction. At any given time point t after the spin echo, the k-space shift $k_y(t)$ due to an MFG G_y in phase encoding direction is given by considering the y-coordinate of Eq. (1), hence

$$\mathrm{k}_y(t) = \gamma\, G_y\;\; t + \; k_{0,y}.$$

The phase-encoding k-space trajectory k_{pe} can be modelled as the traversal of k-space with a constant k-space velocity v, and crossing the center of k-space at a time point τ. This results in a trajectory

$$\mathrm{k}_{pe}(t) = v(t - \tau)$$

In this case, the discrete nature of the phase-encoding blips is neglected, and the finite time of the readout is also not captured in this formula.

The actual center of k-space is traversed at a time point τ_{eff}, when the phase-encoding trajectory plus the MFG-induced shift add to zero. At that moment, the spins in the voxel show no phase-dispersion along the phase-encoding direction and thus the signal is maximal.

$$\mathrm{k}_y(\tau_{eff}) + \mathrm{k}_{pe}(\tau_{eff}) = 0$$

$$\Rightarrow\; \gamma\, G_y\, \tau_{eff} + \; k_{0,y} + v(\tau_{eff} - \tau) = 0$$

We can reformat this

$$\Rightarrow \gamma\, G_y\, \tau_{eff} + v\, \tau_{eff} = v\tau - k_{0,y}$$

$$\Rightarrow \tau_{eff} = \frac{v\tau - k_{0,y}}{v + \gamma\, G_y}$$

$$\Rightarrow \tau_{eff} = \frac{\tau - \frac{k_{0,y}}{v}}{1 + \frac{\gamma\, G_y}{v}}$$

Which is the result reported in Eq. (2) in the manuscript.

The signal dropout out of k-space is modelled separately in our python implementation. But it would manifest as a τ_{eff} that lies outside the sampling time period of the EPI echo train.

Neglecting the blipped nature of the phase-encoding means that the calculated time of k-space center acquisition is slightly different from the one observed in practice. However, it enables a more mathematically elegant result that is not dependent on the number of chosen phase encoding steps. Furthermore, the difference should be smaller than the echo spacing in phase-encoding direction, and thus be well below $1\,ms$.

It is worth noting that the expression for τ_{eff} has a singularity for $\gamma\, G_y = -v$. In this case, the k-space trajectory from the phase-encoding gradient and the one from the MFG cancel out and thus the entire readout happens at the same location of k-space. Thus, the effective center of k-space is never reached. This will lead to the same outcome as signal dropout.

Derivation of Eq. (5)

To investigate the impact of the echo shift on the estimation of R_2', we start with Eq. (4)

$$S_0 \exp(-R_2\, TE) \exp\left(-R_2 \frac{-\frac{\gamma\, G_y}{v}\tau - \frac{\mathrm{k}_{0,y}}{v}}{1+\frac{\gamma\, G_y}{v}}\right) f\left(\frac{\tau - \frac{\mathrm{k}_{0,y}}{v}}{1+\frac{\gamma\, G_y}{v}}\right)$$

and plug in the linear exponential function $f(t) = exp(DBV - R_2'|t|)$.

$$\Rightarrow S_{lin}(\tau) = S_0 \exp(-R_2\, TE) \exp\left(-R_2 \frac{-\frac{\gamma\, G_y}{v}\tau - \frac{\mathrm{k}_{0,y}}{v}}{1+\frac{\gamma\, G_y}{v}} + DBV - R_2' \left|\frac{\tau - \frac{\mathrm{k}_{0,y}}{v}}{1+\frac{\gamma\, G_y}{v}}\right|\right)$$

The absolute value can be divided into two cases for τ:

$$\Rightarrow S_{lin}(\tau) = \begin{cases} S_0 \exp(-R_2\, TE) \exp\left(-R_2 \frac{-\frac{\gamma\, G_y}{v}\tau - \frac{\mathrm{k}_{0,y}}{v}}{1+\frac{\gamma\, G_y}{v}} + DBV - R_2' \frac{\tau - \frac{\mathrm{k}_{0,y}}{v}}{1+\frac{\gamma\, G_y}{v}}\right), \tau \geq \frac{\mathrm{k}_{0,y}}{v} \\ S_0 \exp(-R_2\, TE) \exp\left(-R_2 \frac{-\frac{\gamma\, G_y}{v}\tau - \frac{\mathrm{k}_{0,y}}{v}}{1+\frac{\gamma\, G_y}{v}} + DBV + R_2' \frac{\tau - \frac{\mathrm{k}_{0,y}}{v}}{1+\frac{\gamma\, G_y}{v}}\right), \tau < \frac{\mathrm{k}_{0,y}}{v} \end{cases}$$

Then we can gather the terms in the exponent with τ and the rest.

$$\Rightarrow S_{lin}(\tau) = \begin{cases} S_0 \exp(-R_2\, TE) \exp\left(-\frac{-R_2\frac{\gamma\, G_y}{v}+R_2'}{1+\frac{\gamma\, G_y}{v}}\tau + \frac{R_2+R_2'}{1+\frac{\gamma\, G_y}{v}}\frac{k_{0,y}}{v} + DBV\right), \tau \geq \frac{\mathrm{k}_{0,y}}{v} \\ S_0 \exp(-R_2\, TE) \exp\left(\frac{R_2\frac{\gamma\, G_y}{v}+R_2'}{1+\frac{\gamma\, G_y}{v}}\tau + \frac{R_2-R_2'}{1+\frac{\gamma\, G_y}{v}}\frac{\mathrm{k}_{0,y}}{v} + DBV\right), \tau < \frac{\mathrm{k}_{0,y}}{v} \end{cases} \quad \text{(S1)}$$

The linear-exponential dependence of the signal on τ is retained with the echo shift effect. However, the factor describing the τ dependence, which we describe as the apparent R_2', is affected by the echo shift effect. For $\tau < \frac{\mathrm{k}_{0,y}}{v}$, we see a linear exponential signal rise as a function of τ with an apparent R_2' of $\frac{R_2\frac{\gamma\, G_y}{v}+R_2'}{1+\frac{\gamma\, G_y}{v}}$. For $\tau \geq \frac{\mathrm{k}_{0,y}}{v}$, we see a linear exponential signal decay as a function of τ with an apparent R_2' of $\frac{-R_2\frac{\gamma\, G_y}{v}+R_2'}{1+\frac{\gamma\, G_y}{v}}$.

Derivation of Eq. (6)

We plug in the quadratic exponential $f(t) = exp(-0.3\, DBV\, (\delta\omega\, t)^2)$ into Eq. (4).

$$S_{quad}(\tau) = S_0\, exp(-R_2\, TE)\;\; exp\left(-R_2\, \frac{-\frac{\gamma\, G_y}{v}\tau - \frac{\mathrm{k}_{0,y}}{v}}{1+\frac{\gamma\, G_y}{v}}\right) exp\left(-0.3\, DBV\, \left(\delta\omega\, \frac{\tau - \frac{\mathrm{k}_{0,y}}{v}}{1+\frac{\gamma\, G_y}{v}}\right)^2\right)$$

Then we calculate the apparent DBV with the streamlined qBOLD approach.

$$\overline{DBV} = \ln\left(\frac{S_{lin}(0)}{S_{quad}(0)}\right)$$

We have to account for the two different forms of S_{lin} as presented in Eq. (S1). For the case $0 \geq \frac{\mathrm{k}_{0,y}}{v}$, we use a subscript of +. Furthermore, we use $R_2' = DBV \cdot \delta\omega$.

$$\Rightarrow \overline{DBV_+} = \ln\left(\frac{S_0\, \exp(-R_2\, TE)\, \exp\left(\frac{R_2 + DBV \cdot \delta\omega}{1+\frac{\gamma\, G_y}{v}} \frac{k_{0,y}}{v} + DBV\right)}{S_0\, exp(-R_2\, TE)\;\; exp\left(R_2\, \frac{\frac{\mathrm{k}_{0,y}}{v}}{1+\frac{\gamma\, G_y}{v}}\right) exp\left(-0.3\, DBV\, \left(\delta\omega\, \frac{\frac{\mathrm{k}_{0,y}}{v}}{1+\frac{\gamma\, G_y}{v}}\right)^2\right)}\right)$$

$$\Rightarrow \overline{DBV_+} = \frac{R_2 + DBV \cdot \delta\omega}{1+\frac{\gamma\, G_y}{v}} \frac{k_{0,y}}{v} + DBV - R_2\, \frac{\frac{\mathrm{k}_{0,y}}{v}}{1+\frac{\gamma\, G_y}{v}} + 0.3\, DBV\, \left(\delta\omega\, \frac{\frac{\mathrm{k}_{0,y}}{v}}{1+\frac{\gamma\, G_y}{v}}\right)^2$$

$$\Rightarrow \overline{DBV_+} = DBV\left(1 + \delta\omega \frac{\frac{k_{0,y}}{v}}{1+\frac{\gamma\, G_y}{v}} + 0.3\left(\delta\omega\, \frac{\frac{\mathrm{k}_{0,y}}{v}}{1+\frac{\gamma\, G_y}{v}}\right)^2\right)$$

For the case $0 < \frac{\mathrm{k}_{0,y}}{v}$, we use a subscript of -.

$$\Rightarrow \overline{DBV_-} = \ln\left(\frac{S_0\, \exp(-R_2\, TE)\, \exp\left(\frac{R_2 - DBV \cdot \delta\omega}{1+\frac{\gamma\, G_y}{v}} \frac{k_{0,y}}{v} + DBV\right)}{S_0\, exp(-R_2\, TE)\;\; exp\left(R_2\, \frac{\frac{\mathrm{k}_{0,y}}{v}}{1+\frac{\gamma\, G_y}{v}}\right) exp\left(-0.3\, DBV\, \left(\delta\omega\, \frac{\frac{\mathrm{k}_{0,y}}{v}}{1+\frac{\gamma\, G_y}{v}}\right)^2\right)}\right)$$

$$\Rightarrow \overline{DBV_-} = \frac{R_2 - DBV \cdot \delta\omega}{1+\frac{\gamma\, G_y}{v}} \frac{k_{0,y}}{v} + DBV - R_2\, \frac{\frac{\mathrm{k}_{0,y}}{v}}{1+\frac{\gamma\, G_y}{v}} + 0.3\, DBV\, \left(\delta\omega\, \frac{\frac{\mathrm{k}_{0,y}}{v}}{1+\frac{\gamma\, G_y}{v}}\right)^2$$

$$\Rightarrow \overline{DBV_-} = DBV\left(1 - \delta\omega \frac{\frac{k_{0,y}}{v}}{1+\frac{\gamma\, G_y}{v}} + 0.3\left(\delta\omega\, \frac{\frac{\mathrm{k}_{0,y}}{v}}{1+\frac{\gamma\, G_y}{v}}\right)^2\right)$$

We can summarize these two formulas, which results in Eq. (6) from the main text.

$$\Rightarrow \widetilde{DBV} = \begin{cases} DBV\left(1+\delta\omega\frac{\frac{k_{0,y}}{v}}{1+\frac{\gamma\, G_y}{v}}+0.3\left(\delta\omega\;\frac{\frac{\mathrm{k}_{0,y}}{v}}{1+\frac{\gamma\, G_y}{v}}\right)^2\right), 0 \geq \frac{\mathrm{k}_{0,y}}{v} \\ DBV\left(1-\delta\omega\frac{\frac{k_{0,y}}{v}}{1+\frac{\gamma\, G_y}{v}}+0.3\left(\delta\omega\;\frac{\frac{\mathrm{k}_{0,y}}{v}}{1+\frac{\gamma\, G_y}{v}}\right)^2\right), 0 < \frac{\mathrm{k}_{0,y}}{v} \end{cases}$$

Note that Eq. (6) assumes that all images have been acquired with the same $\mathrm{k}_{0,y}$. However, if images from multiple $\mathrm{k}_{0,y}$ are combined before the signal trajectory is fitted or if different $\mathrm{k}_{0,y}$ are applied at different τ to account for the varying magnitude of the MFG-induced dephasing, the estimation of DBV would become even more difficult.

For the simulated parameters (described in section 3.1.2), a $k_{0,y}$ corresponding to a shift of a single k-space line, $\mathrm{k}_{0,y,single} = \frac{2\pi}{n_y\,\Delta y} = 0.028\,\frac{rad}{mm}$, would already result in $\widetilde{DBV} \approx 1.05\,DBV$, even when $G_y = 0$. As a result, it is of interest to validate a vanishing $\mathrm{k}_{0,y}$ value in practice to ensure accurate DBV results.

For the volunteer images acquired with $\tau = 0\;ms$ and parallel imaging factor 2 in this work, the k-space shift, as determined using the simulated signal dropout method, took a median value across the whole brain of $k_{0,y} \approx 0.028\,\frac{rad}{mm}$, regardless of phase-encoding direction.

Derivation of Eq. (7)

We use the expression for the echo shift for the image with reversed phase encoding direction of $\tau_{eff,rev}$. We insert the formulas of the linear exponential signal model for positive τ and τ_{eff} to calculate $\ln\left(\frac{S_{lin}(\tau)}{S_{lin,rev}(\tau)}\right)$. The terms can then be simplified.

$$\Rightarrow \ln\left(\frac{S_{lin}(\tau)}{S_{lin,rev}(\tau)}\right) = \ln\left(\frac{S_0\,\exp(-R_2\,TE)\,\exp\left(-R_2\left(\tau_{eff}-\tau\right)+DBV-R_2'\,\tau_{eff}\right)}{S_0\,\exp(-R_2\,TE)\,\exp\left(-R_2\left(\tau_{eff,rev}-\tau\right)+DBV-R_2'\,\tau_{eff,rev}\right)}\right)$$

$$\Rightarrow \ln\left(\frac{S_{lin}(\tau)}{S_{lin,rev}(\tau)}\right) = \ln\left(\exp\left(-R_2\left(\tau_{eff}-\tau\right)-R_2'\,\tau_{eff}+R_2\left(\tau_{eff,rev}-\tau\right)+R_2'\,\tau_{eff,rev}\right)\right)$$

$$\Rightarrow \ln\left(\frac{S_{lin}(\tau)}{S_{lin,rev}(\tau)}\right) = -(R_2'+R_2)\cdot\left(\tau_{eff}(\tau)-\tau_{eff,rev}(\tau)\right)$$

This is the form of Eq. (7) presented in the main text. For negative τ, the dependence on R_2' would flip its sign.

Derivation of Eq. (8)

We use the expression $\tau_{eff} = \frac{\tau - \frac{k_{0,y}}{v}}{1+\frac{\gamma G_y}{v}}$ from Eq. (2). For the reverse phase-encoding direction image, we can define $\tau_{eff,rev} = \frac{\tau - \frac{k_{0,y,rev}}{v}}{1-\frac{\gamma G_y}{v}}$, assuming that the reverse phase-encoding direction results in an opposite MFG G_y. While the opposite G_y is expected for the reverse phase-encoding direction, $k_{0,y}$ may in general not show such predictable behavior, e.g. if it arises due to eddy currents.

Inserting these expressions in Eq. (7) yields:

$$\Rightarrow \ln\left(\frac{S_{lin}(\tau)}{S_{lin,rev}(\tau)}\right) = -(R_2' + R_2) \cdot \left(\frac{\tau - \frac{k_{0,y}}{v}}{1 + \frac{\gamma G_y}{v}} - \frac{\tau - \frac{k_{0,y,rev}}{v}}{1 - \frac{\gamma G_y}{v}}\right)$$

$$\Rightarrow \ln\left(\frac{S_{lin}(\tau)}{S_{lin,rev}(\tau)}\right) = -(R_2' + R_2) \cdot \left(\frac{\tau}{1 + \frac{\gamma G_y}{v}} - \frac{\tau}{1 - \frac{\gamma G_y}{v}} - \frac{\frac{k_{0,y}}{v}}{1 + \frac{\gamma G_y}{v}} + \frac{\frac{k_{0,y,rev}}{v}}{1 - \frac{\gamma G_y}{v}}\right)$$

$$\Rightarrow \ln\left(\frac{S_{lin}(\tau)}{S_{lin,rev}(\tau)}\right) = -(R_2' + R_2) \cdot \left(\frac{\tau\left(1 - \frac{\gamma G_y}{v}\right) - \tau\left(1 + \frac{\gamma G_y}{v}\right)}{1 - \left(\frac{\gamma G_y}{v}\right)^2} - \frac{\frac{k_{0,y}}{v}\left(1 - \frac{\gamma G_y}{v}\right) - \frac{k_{0,y,rev}}{v}\left(1 + \frac{\gamma G_y}{v}\right)}{1 - \left(\frac{\gamma G_y}{v}\right)^2}\right)$$

$$\Rightarrow \ln\left(\frac{S_{lin}(\tau)}{S_{lin,rev}(\tau)}\right) = -(R_2' + R_2) \cdot \left(\frac{-2\tau\frac{\gamma G_y}{v}}{1 - \left(\frac{\gamma G_y}{v}\right)^2} - \frac{\left(\frac{k_{0,y}}{v} - \frac{k_{0,y,rev}}{v}\right) - \left(\frac{k_{0,y}}{v} + \frac{k_{0,y,rev}}{v}\right)\frac{\gamma G_y}{v}}{1 - \left(\frac{\gamma G_y}{v}\right)^2}\right)$$

For high values of τ, which is where the linear exponential expression applies, we can assume that $\frac{k_{0,y}}{v}$ and $\frac{k_{0,y,rev}}{v}$ are neglible compared to τ. Then the formula simplifies to Eq. (8) from the main text:

$$\Rightarrow \ln\left(\frac{S_{lin}(\tau)}{S_{lin,rev}(\tau)}\right) = 2(R_2' + R_2)\tau \frac{\frac{\gamma G_y}{v}}{1 - \left(\frac{\gamma G_y}{v}\right)^2}$$

It's worth noting that this relationship is approximately linear for small values of $\frac{\gamma G_y}{v}$. For negative τ, the flipped sign for the R_2' dependence from Eq. (7) would carry over to this equation as well.

Additional Simulated Signal Curves

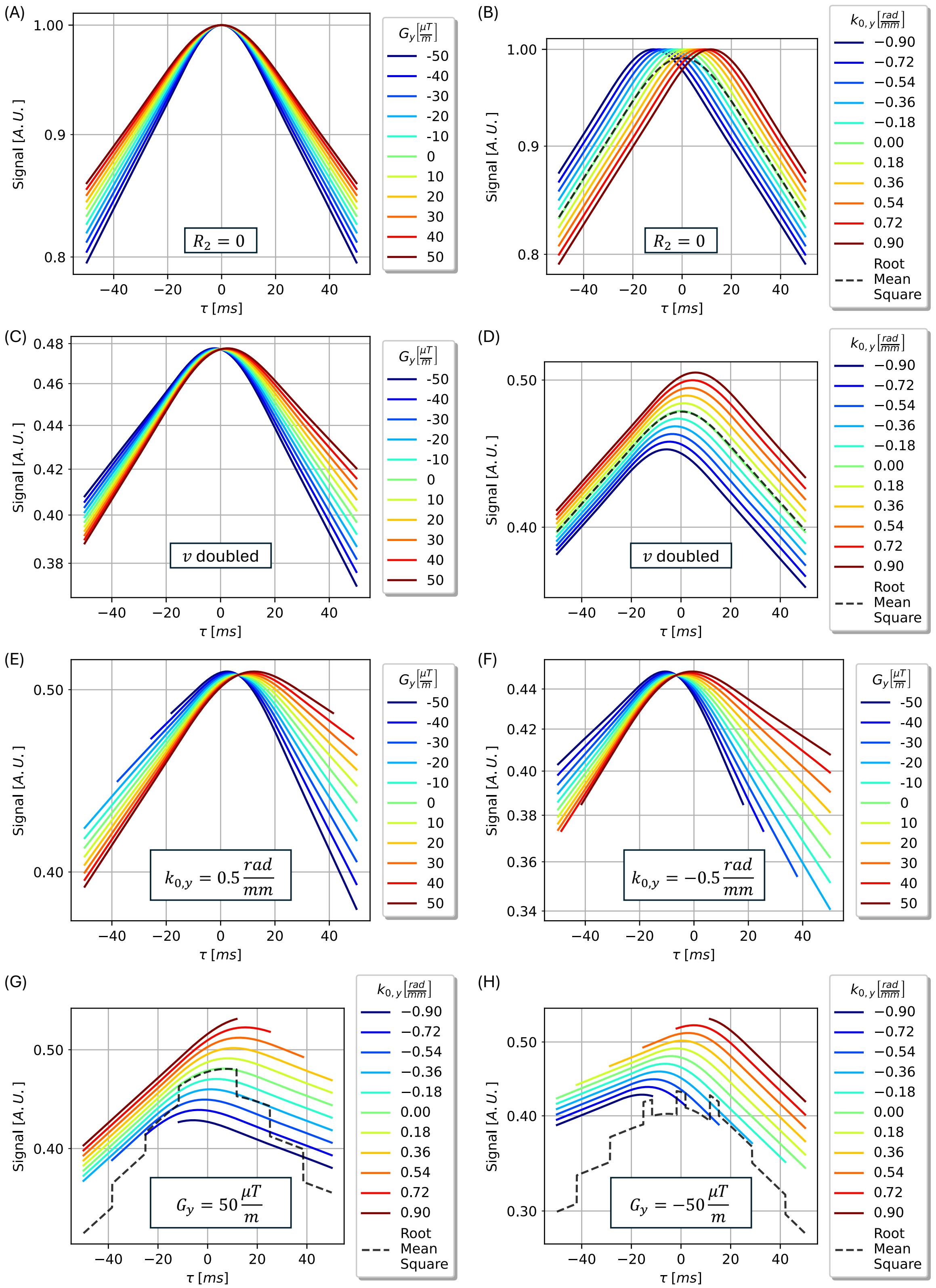

Figure S1. Simulated ASE signal trajectories for different acquisition and tissue settings. Simulation parameters are identical to those presented in the main text unless specified. Gaps in simulated lines indicate signal dropout has occurred. (A,B) simulate no R_2 decay. (C,D) simulate a doubled k-space velocity. (E,F) show the impact of different G_y given a non-zero $k_{0,y}$. (G,H) show the impact of different $k_{0,y}$ given a non-zero G_y. The discontinuous

nature of the root mean square curves is a result of the signal dropout in the simulated curves. It would likely not be observed in reality, as signal dropout would not occur so abruptly and all images have a noise contribution.

Images at intermediate τ

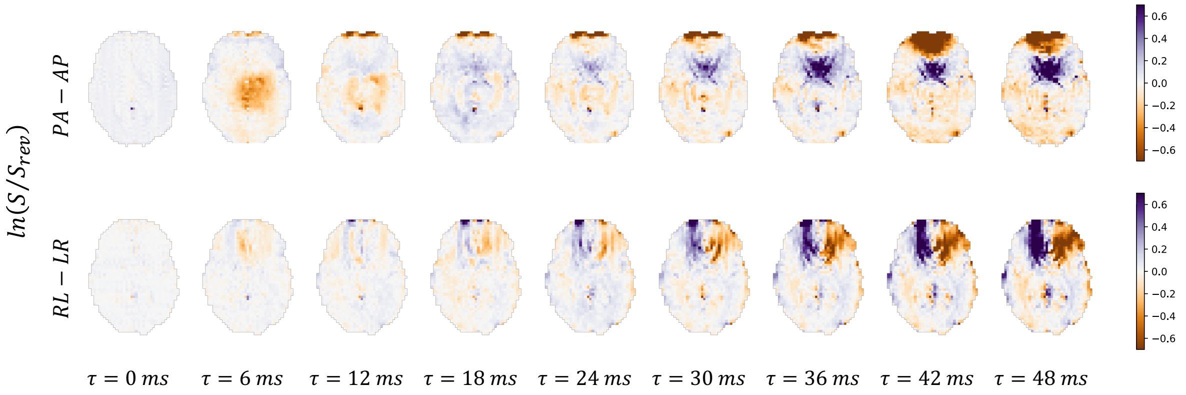

Figure S2. $ln(S/S_{rev})$ for all acquired τ with parallel imaging factor 2 of volunteer 2. The slice is the same as shown in Figure 7. A gradual development of the signal towards the pattern explained by the echo shift is visible. However, some unexplained anomalies exist for low τ values, e.g. the orange area for PA-AP $\tau = 6\,ms$.

Correction of Echo Shift Effect in Post-Processing

Numerical correction

One option for correcting the echo shift effect is to use a correction factor for the measured signal. Conventionally, it is assumed that the measured signals take the form $S(\tau)$ from Eq. (3) and thus qBOLD parameters are acquired by fitting the function $f(\tau)$ to the signals. In that case, this is the only dependence on τ. With the echo-shift effect, the measured signals take form $S_{shift}(\tau)$ from Eq. (4) instead. To still facilitate an accurate fit, τ_{eff} can be estimated, for example with the simulated signal dropout method described in this work, or calculated as $\tau_{eff} = \frac{\tau - \frac{k_{0,y}}{v}}{1 + \frac{\gamma G_y}{v}}$ based on a measurement of G_y using a separate B_0 map. Then, the dependence of the signal on τ_{eff} via R_2 can be compensated by multiplying the signal with a correction factor

$$C(\tau) = \exp\left(R_2\left(\tau_{eff} - \tau\right)\right). \qquad \text{(S2)}$$

This would require an estimation of R_2.

After multiplication of this factor, the corrected signal would depend on τ_{eff} only via $f(\tau_{eff})$, and so a correct fit of f could be achieved in a similar way to the conventional qBOLD approach but by taking into account the τ_{eff} values.

Combining data with opposing phase encoding directions

Let $S(\tau)$ describe the acquired signal and $S_{rev}(\tau)$ describe the signal acquired with reversed phase-encoding direction. In this case, the geometric mean of the two signals, as defined in Eq. (4), leads to the following expression:

$$\sqrt{S(\tau)\, S_{rev}(\tau)} = \sqrt{S_0 \exp(-R_2\, TE) \exp\left(-R_2\left(\tau_{eff} - \tau\right)\right) f\left(\tau_{eff}\right)} \cdot \sqrt{S_0 \exp(-R_2\, TE) \exp\left(-R_2\left(\tau_{eff,rev} - \tau\right)\right) f\left(\tau_{eff,rev}\right)}$$

$$= S_0 \exp(-R_2\, TE) \sqrt{\exp\left(-R_2\left(\tau_{eff} + \tau_{eff,rev} - 2\tau\right)\right) f\left(\tau_{eff}\right)\, f\left(\tau_{eff,rev}\right)}$$

$$= S_0 \exp(-R_2\, TE) \exp\left(-R_2\left(\frac{\tau_{eff}+\tau_{eff,rev}}{2} - \tau\right)\right) \sqrt{f\left(\tau_{eff}\right)\, f\left(\tau_{eff,rev}\right)} \qquad \text{(S3)}$$

The R_2 dependence can be investigated by using $\tau_{eff} = \frac{\tau - \frac{k_{0,y}}{v}}{1+\frac{\gamma\, G_y}{v}}$ and $\tau_{eff,rev} = \frac{\tau - \frac{k_{0,y,rev}}{v}}{1-\frac{\gamma\, G_y}{v}}$.

We can calculate the addition $\tau_{eff} + \tau_{eff,rev}$:

$$\tau_{eff} + \tau_{eff,rev} = \frac{\tau - \frac{k_{0,y}}{v}}{1 + \frac{\gamma\, G_y}{v}} + \frac{\tau - \frac{k_{0,y,rev}}{v}}{1 - \frac{\gamma\, G_y}{v}}$$

$$= \frac{\left(\tau - \frac{k_{0,y}}{v}\right)\left(1 - \frac{\gamma\, G_y}{v}\right)}{1 - \left(\frac{\gamma\, G_y}{v}\right)^2} + \frac{\left(\tau - \frac{k_{0,y,rev}}{v}\right)\left(1 + \frac{\gamma\, G_y}{v}\right)}{1 - \left(\frac{\gamma\, G_y}{v}\right)^2}$$

$$= \frac{\tau - \frac{k_{0,y}}{v} - \frac{\gamma\, G_y}{v}\tau + \frac{\gamma\, G_y}{v}\frac{k_{0,y}}{v} + \tau - \frac{k_{0,y,rev}}{v} + \frac{\gamma\, G_y}{v}\tau - \frac{\gamma\, G_y}{v}\frac{k_{0,y,rev}}{v}}{1 - \left(\frac{\gamma\, G_y}{v}\right)^2}$$

$$= \frac{2\tau - \frac{k_{0,y} + k_{0,y,rev}}{v} + \frac{\gamma\, G_y}{v}\frac{k_{0,y} - k_{0,y,rev}}{v}}{1 - \left(\frac{\gamma\, G_y}{v}\right)^2}$$

If the terms $k_{0,y}$ and $k_{0,y,rev}$ can be neglected, e.g. for large $|\tau|$, then we can simplify the expression to

$$\tau_{eff} + \tau_{eff,rev} \approx \frac{2\tau}{1-\left(\frac{\gamma\, G_y}{v}\right)^2}. \qquad \text{(S4)}$$

The R_2 dependence in Eq. (S3) can thus be written as

$$\exp\left(-R_2\left(\frac{\tau_{eff} + \tau_{eff,rev}}{2} - \tau\right)\right) = \exp\left(-R_2\, \tau\left(\frac{1}{1 - \left(\frac{\gamma\, G_y}{v}\right)^2} - 1\right)\right).$$

Compared to the conventional relationship in Eq. (4), the dependence on $\frac{\gamma\, G_y}{v}$ is now of the second order, which may be neglible for many brain voxels.

The dependence of the geometric mean signal on f varies with the choice of f. For the linear exponential regime, we can investigate $f(t) = exp(DBV - R_2'|t|)$.

$$\sqrt{f(\tau_{eff})\ f(\tau_{eff,rev})} = \sqrt{\exp(DBV - R_2'|\tau_{eff}|)\ \exp(DBV - R_2'|\tau_{eff,rev}|)}$$

$$= \exp\left(DBV - R_2{}' \left(\frac{|\tau_{eff}| + |\tau_{eff,rev}|}{2} \right) \right)$$

For $\tau_{eff}, \tau_{eff,rev} > 0$, we can insert the expression from Eq. (S4) to obtain

$$\sqrt{f(\tau_{eff})\ f(\tau_{eff,rev})} = \exp\left(DBV - R_2{}' \frac{\tau}{1 - \left(\frac{\gamma\, G_y}{v}\right)^2} \right).$$

Thus, the dependence on $\frac{\gamma\, G_y}{v}$ is also taken to the second order for this term.

For the quadratic exponential regime, we use $f(t) = exp(-0.3\ DBV\ \delta\omega^2\ t^2)$. This yields

$$\sqrt{f(\tau_{eff})\ f(\tau_{eff,rev})} = \sqrt{\exp(-0.3\ DBV\ \delta\omega^2\ \tau_{eff}{}^2)\exp(-0.3\ DBV\ \delta\omega^2\ \tau_{eff,rev}{}^2)}$$

$$= \exp\left(-0.3\ DBV\ \delta\omega^2 \left(\frac{\tau_{eff}{}^2 + \tau_{eff,rev}{}^2}{2} \right) \right).$$

If we can once again assume that $k_{0,y}$ and $k_{0,y,rev}$ can be neglected, which is not necessarily trivial for small τ values, then we can further simplify this expression. We can write

$$\tau_{eff}{}^2 + \tau_{eff,rev}{}^2 \approx \frac{\tau^2}{\left(1 + \frac{\gamma\, G_y}{v}\right)^2} + \frac{\tau^2}{\left(1 - \frac{\gamma\, G_y}{v}\right)^2}$$

$$= \tau^2 \left(\frac{\left(1 - \frac{\gamma\, G_y}{v}\right)^2}{\left(\left(1 + \frac{\gamma\, G_y}{v}\right)\left(1 - \frac{\gamma\, G_y}{v}\right)\right)^2} + \frac{\left(1 + \frac{\gamma\, G_y}{v}\right)^2}{\left(\left(1 + \frac{\gamma\, G_y}{v}\right)\left(1 - \frac{\gamma\, G_y}{v}\right)\right)^2} \right)$$

$$= \tau^2 \left(\frac{1 - 2\frac{\gamma\, G_y}{v} + \left(\frac{\gamma\, G_y}{v}\right)^2 + 1 + 2\frac{\gamma\, G_y}{v} + \left(\frac{\gamma\, G_y}{v}\right)^2}{\left(1 - \left(\frac{\gamma\, G_y}{v}\right)^2\right)^2} \right)$$

$$= 2\tau^2 \frac{1 + \left(\frac{\gamma\, G_y}{v}\right)^2}{\left(1 - \left(\frac{\gamma\, G_y}{v}\right)^2\right)^2}.$$

Substituting this into the geometric mean expression for f thus leads to:

$$\sqrt{f(\tau_{eff})\ f(\tau_{eff,rev})} = \exp\left(-0.3\ DBV\ \delta\omega^2\tau^2 \frac{1 + \left(\frac{\gamma\, G_y}{v}\right)^2}{\left(1 - \left(\frac{\gamma\, G_y}{v}\right)^2\right)^2}\right).$$

Once again, the dependences on $\frac{\gamma\, G_y}{v}$ are at least of second order. As a result, this geometric mean of signals may for most voxels give a close estimate of the signal without the echo-shift effect.

When this approach is used, the difference in geometric distortions between the images needs to be accounted for. Additionally, the equation presented here does not account for the fact that the two images show different sensitivity to through-plane dephasing due to the different τ_{eff} values. This may have to be corrected for in an additional step.
